# James Truman, D.D.S., L.L.D.

**Published:** 1915-02

**Authors:** 


### OBITUARY.

JAMES TRUMAN, D.D.S., L.L.D.—Dr. Truman died at his home in Philadelphia, November 26, 1914, in his eighty-eighth year. He was born at Abington, Pa., November 22, 1826. He lived practically all his life in Philadelphia. He graduated from the old Philadelphia Dental College in 1854. In 1864 he began his teaching career as Demonstrator of Operative Dentistry in the Pennsylvania Dental College. He was one of the first to advocate bleaching of discolored teeth. He was Professor of Dental Physiology and Operative Dentistry in the Pennsylvania Dental College from 1866 to 1896. He was among the first to advocate the education of women for dentistry. His first editorial work was on the *Dental Times*, of which he was editor for four years. From 1890 to 1905 he was editor of the *International Journal*, where he made a reputation as a versatile and conservative editorial commentator on all current dental events. He became associated with the Dental Department of the University of Pennsylvania in 1882, and served that school until he retired from teaching work in 1909. He served the school as its Secretary and Dean and was Professor of Dental Pathology and Therapeutics. He was a constant attendant of the meetings of the National Dental Association and other prominent organizations. He was a sincere and conscientious man, and had a commanding and determined disposition which brought him into strenuous debates with his confreres in dental meetings. His mind was logical and his speech vigorous and sometimes dogmatic. He was respected by all and loved by those who knew him well. He was one of the giants of his day. He did his work well and the profession is better because of his life.

